# Multi-anion/cation engineering enables fast ion transport and stable interfaces in Zr-based halide electrolytes for all-solid-state batteries

**DOI:** 10.1039/d6sc04008j

**Published:** 2026-06-16

**Authors:** Xuele Xu, Zihan Yan, Jiachen Ying, Linghao Deng, Mingxing Liu, Yifei Xu, Yao Liu, Yiwen Zhang, Yizhou Zhu, Yongyao Xia, Yonggang Wang

**Affiliations:** a Department of Chemistry, Shanghai Key Laboratory of Molecular Catalysis and Innovative Materials, Institute of New Energy, Laboratory of Advanced Materials, iChEM (Collaborative Innovation Center of Chemistry for Energy Materials), Fudan University Shanghai 200433 China yyxia@fudan.edu.cn ygwang@fudan.edu.cn; b Department of Materials Science and Engineering, Westlake University Hangzhou Zhejiang 310030 China zhuyizhou@westlake.edu.cn; c State Key Laboratory of Molecular Engineering of Polymers, Department of Macromolecular Science, Fudan University Shanghai China; d Shanghai Institute of Applied Physics, Chinese Academy of Sciences Shanghai 201800 China; e Mathematics and Science College, Shanghai Normal University Shanghai 200234 China

## Abstract

The Zr-based halide solid-state electrolyte (SE) Li_2_ZrCl_6_ (LZC) has emerged as a promising candidate for all-solid-state lithium metal batteries (ASSLBs) due to the natural abundance and low cost of zirconium. However, its relatively low ionic conductivity and insufficient interfacial stability severely limit its practical application. Herein, we propose a synergistic multi-anion and cation co-doping strategy to construct a highly amorphous halide electrolyte, Li_2.25_Zr_0.75_Al_0.25_Cl_4.2_O_0.8_F_0.2_. The incorporation of O^2−^ induces substantial amorphization, facilitating Li^+^ transport and improving mechanical deformability. Meanwhile, Al^3+^ substitution increases Li^+^ concentration *via* charge compensation, further promoting ion transport. In addition, F^−^ incorporation enhances oxidative stability and enables the formation of a robust F-rich cathode–electrolyte interphase (CEI), effectively suppressing interfacial side reactions. As a result, the optimized electrolyte exhibits a high ionic conductivity of 1.12 × 10^−3^ S cm^−1^ with reduced activation energy. When paired with a LiNi_0.90_Co_0.05_Mn_0.05_O_2_ cathode, the assembled ASSLB delivers excellent rate capability and long-term stability, retaining ∼70% of its initial capacity after 500 cycles at 0.5C. This work demonstrates that synergistic multi-anion and cation engineering enables amorphization that effectively couples fast ion transport with stable interfaces in halide solid electrolytes.

## Abstract 2

Synergistic multi-anion/cation engineering induces amorphization and Li^+^ enrichment, enabling fast ion transport and stable interfaces in Zr-based halide electrolytes for high-performance solid-state batteries.

## Graphical abstract

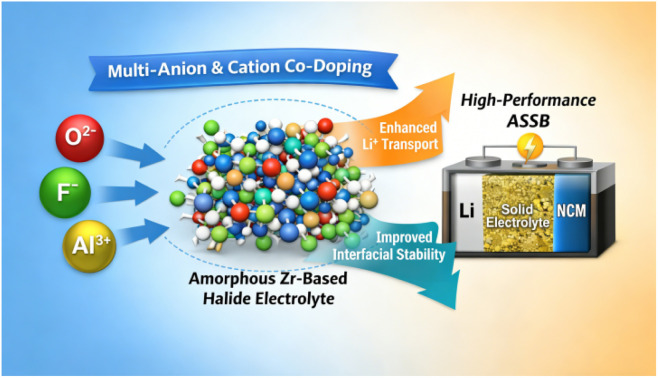

## Introduction

The rapid growth of electric vehicles and large-scale energy storage systems has led to a dramatic increase in the demand for lithium-ion batteries (LIBs). However, conventional LIBs based on liquid electrolytes are approaching their theoretical energy density limits and continue to suffer from critical safety concerns.^1,2^ In this situation, ASSLBs have emerged as a promising next-generation technology, owing to the use of safe solid electrolytes and the integration of high-capacity lithium metal anodes with high-energy cathodes, which together offer the potential to surpass the energy density limits of current LIBs.^3–6^

Inorganic solid electrolytes, including oxides, sulfides, and halides, have been extensively investigated for ASSLBs. Oxide electrolytes, such as NASICON-type Li_1.5_Al_0.5_Ge_1.5_(PO_4_)_3_ (ref. 7) and garnet-type Li_7_La_3_Zr_2_O_12_,^8^ typically require high-temperature sintering above 1000 °C.^9,10^ Although they exhibit wide electrochemical stability windows and high mechanical strength, their poor interfacial contact with cathode materials often necessitates the use of liquid additives to improve wettability.^11,12^ Sulfide electrolytes, such as argyrodite Li_6−*a*_PS_5−*a*_X_1+*a*_ (X = Cl, Br, I)^13^ and Li_10_GeP_2_S_12_,^14^ possess exceptionally high ionic conductivity (≥10^−2^ S cm^−1^) and excellent deformability.^15^ However, their limited oxidative stability (typically < 2.5 V *versus* Li/Li^+^) severely hinders their compatibility with high-voltage cathodes and practical applications.^16,17^ In comparison, halide solid electrolytes combine the advantages of both oxides and sulfides, exhibiting relatively high ionic conductivities (10^−4^–10^−3^ S cm^−1^ at room temperature), wide oxidative stability windows (∼4.0 V *versus* Li/Li^+^), and favorable mechanical deformability.^18,19^ These materials are generally represented by the formula Li–M–X (M = Y, Zr, In, Yb, *etc.*; X = F, Cl, Br, I),^18^ with typical examples including Li_3_YCl_6_,^20^ Li_3_InCl_6_,^21^ and Li_3_ScCl_6_.^22^ Nevertheless, many high-performance halide electrolytes rely on expensive group IIIB elements, resulting in high material costs and limiting their large-scale application.^23^ Therefore, the development of cost-effective halide electrolytes based on earth-abundant elements is highly desirable.

Recently, Zr-based halide electrolytes have attracted increasing attention due to the high natural abundance and low cost of zirconium.^24–26^ In 2021, Ma *et al.* first reported Li_2_ZrCl_6_ (LZC),^24^ although its room-temperature ionic conductivity was relatively low (∼0.4 mS cm^−1^).^27^ Subsequent studies have employed elemental doping strategies to enhance its ionic conductivity. For example, Al^3+^ substitution for Zr^4+^ increased the ionic conductivity to 1.13 mS cm^−1^,^23^ while partial substitution with Cu^2+^ yielded a conductivity of 0.75 mS cm^−1^ and a reduced activation energy of 0.268 eV.^28^ In addition, O^2−^ incorporation enabled the synthesis of Li_3_Zr_0.75_OCl_4_ with an ionic conductivity of 1.35 mS cm^−1^ at room temperature.^29^ Despite these advances, most of the ASSLBs based on modified LZC electrolytes still exhibit limited cycling stability, typically below 500 cycles (Table S1).^18,22,23,25,28,30–35^ This limitation arises because, beyond ionic conductivity, the stability of the electrode/electrolyte interface and the suppression of interfacial side reactions play a critical role in determining long-term battery performance.^18,27^ Current modification strategies for LZC primarily rely on single cation or anion doping, which, although effective in enhancing ionic conductivity, are insufficient to simultaneously improve interfacial stability and mitigate parasitic reactions.

Herein, we propose a multi-anion and cation co-doping strategy to synergistically enhance the overall performance of LZC-based solid electrolytes. Specifically, a Li_2.25_Zr_0.75_Al_0.25_Cl_4.2_O_0.8_F_0.2_ electrolyte was synthesized *via* a one-step mechanochemical route. Structural analysis and theoretical calculation reveal that O^2−^ incorporation significantly increases the amorphous fraction (∼74%), thereby improving mechanical deformability and enhancing ionic conductivity to 1.12 × 10^−3^ S cm^−1^, substantially higher than that of pristine LZC (3.97 × 10^−4^ S cm^−1^). Meanwhile, F^−^ incorporation contributes to improved oxidative stability. Furthermore, Al^3+^ substitution for Zr^4+^ increases the Li^+^ concentration *via* charge compensation, leading to further enhancement of Li^+^ transport. When paired with a LiNi_0.90_Co_0.05_Mn_0.05_O_2_ (NCM90) cathode, the resulting ASSLB (Li–In|Li_6_PS_5_Cl|halide SE|NCM90) delivers excellent rate capability and long-term cycling stability within a voltage window of 2.6–4.3 V (*versus* Li/Li^+^), achieving a capacity retention of ∼70% after 500 cycles at 0.5C. This superior performance is attributed to the synergistic effects of enhanced ionic conductivity and improved deformability associated with the highly amorphous structure. In addition, interfacial characterization reveals the formation of a fluorine-rich CEI, which effectively suppresses interfacial side reactions. This work provides new insights into the design of cost-effective, high-performance halide solid electrolytes and offers guidance for cathode–electrolyte interface engineering.

## Results and discussion

**Fig. 1:**
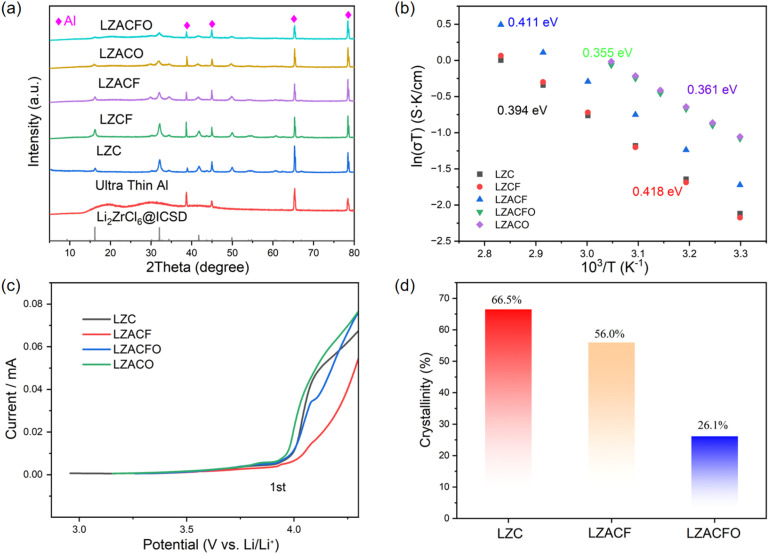
(a) X-ray diffraction patterns of LZC, LZCF, LZACF, LZACO, and LZACFO. (b) The Arrhenius plots of ionic conductivities for LZC, LZCF, LZACF, LZACO, and LZACFO. (c) LSV curves of LZC, LZACF, LZACO, and LZACFO. (d) Quantitative analysis of the crystallinity of LZC, LZACF, and LZACFO.

Li_2_ZrCl_6_ (LZC) was synthesized *via* a one-step mechanical ball-milling process using stoichiometric LiCl and ZrCl_4_, followed by low-temperature annealing. Li_2+*x*_Zr_1−*x*_Al_*x*_Cl_5.8_F_0.2_ (*x* = 0.00 and 0.25; LZCF and LZACF, respectively) was prepared by introducing stoichiometric amounts of LiF and AlCl_3_ into the precursors, where low-valent Al^3+^ partially replaces Zr^4+^ to increase the Li^+^ concentration for charge compensation. Subsequently, Li_2_O was incorporated to synthesize Li_2.25_Zr_0.75_Al_0.25_Cl_4.4−*x*_F_*x*_O_0.8_ (*x* = 0.0 and 0.2; LZACO and LZACFO, respectively), introducing O^2−^ to reduce crystallinity. The X-ray diffraction (XRD) patterns of the as-prepared samples are shown in Fig. 1a. To prevent air exposure, the sample holder was covered with an ultrathin Al layer during measurements. All diffraction peaks are consistent with the trigonal structure of Li_2_ZrCl_6_ (space group *P*3̄*m*1, PDF No. 04-028-3319), and no peaks corresponding to raw materials or impurities are observed, confirming successful incorporation of the dopants without altering the phase or crystal structure.

The ionic conductivity and activation energy of these solid electrolytes were determined by electrochemical impedance spectroscopy (EIS), with results shown in Fig. 1b (impedance spectra at different temperatures are presented in Fig. S1). As shown in Fig. 1b, LZC exhibits a conductivity of 3.97 × 10^−4^ S cm^−1^ at 30 °C. The incorporation of F^−^ (yielding LZCF) results in a slight decrease of ionic conductivity to 3.75 × 10^−4^ S cm^−1^. This diminution is ascribed to enhanced electrostatic interactions between the anionic sublattice and mobile Li^+^ species, which collectively impede long-range charge transport. Consequently, LZCF was precluded from subsequent electrochemical evaluation. In contrast, LZACF exhibits a higher conductivity of 5.89 × 10^−4^ S cm^−1^ at 30 °C. This enhancement is attributed to Al^3+^ substitution for Zr^4+^, which increases the Li^+^ concentration in the lattice *via* charge compensation, thereby facilitating Li^+^ transport. Amorphous solid-state electrolytes (SEs) lack long-range periodic atomic order and instead form a distinctive glass-like network, which weakens the interaction between the anion framework and Li^+^, thereby promoting high ionic conductivity.^36,37^ To further enhance conductivity, O^2−^ was introduced to form LZACO and LZACFO, as oxygen incorporation generally increases the amorphous fraction.^6,38^ As shown in the Arrhenius plot of total ionic conductivity (Fig. 1b), LZACO and LZACFO exhibit significantly higher conductivities than LZC, reaching 1.15 × 10^−3^ and 1.12 × 10^−3^ S cm^−1^, respectively, accompanied by lower activation energies (0.361 eV and 0.355 eV, *versus* 0.394 eV for LZC), highlighting the beneficial effect of oxygen on Li^+^ transport.

Electrochemical stability was assessed by linear sweep voltammetry (LSV) using Li–In|Li_6_PS_5_Cl|halide SE|halide SE-Super P (7 : 3 by weight) cells. In this configuration, a Li_6_PS_5_Cl interlayer was used to suppress interfacial reactions, and conductive carbon (Super P) was incorporated to improve electronic contact between the electrolyte particles. The measured potentials *versus* Li–In were converted to the Li/Li^+^ scale by applying a correction of 0.62 V, corresponding to the potential difference between the Li–In alloy and Li metal.^39^ LSV results (Fig. 1c and the enlargement of Fig. 1c and S2) indicate that LZC and LZACF exhibit oxidation limits of 3.99 and 4.07 V *versus* Li/Li^+^, respectively, demonstrating the stabilizing effect of F^−^ incorporation. LZACO shows a slightly lower onset at 3.95 V, suggesting that oxygen alone marginally reduces oxidative stability, whereas co-doping with F^−^ in LZACFO restores the anodic limit to 3.98 V. Considering both conductivity and electrochemical stability, LZACF and LZACFO were selected for further evaluation. The enlarged XRD patterns of LZC, LZACF and LZACFO (Fig. S3) show that the diffraction peaks broaden progressively from LZC to LZACF and LZACFO, indicating an increasing amorphous fraction in the order LZC < LZACF < LZACFO. The peak broadening in LZACF reflects enhanced amorphization due to Al^3+^ and F^−^ incorporation, while the further broadening in LZACFO indicates additional disorder from O^2−^. The estimated crystallinity decreases from 66.5% for LZC to 56.0% for LZACF and 26.1% for LZACFO (Fig. 1d), confirming the progressive increase in amorphous content.

**Fig. 2:**
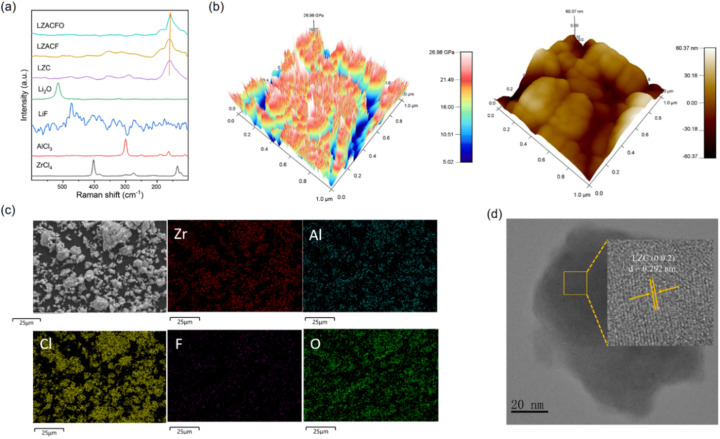
(a) The Raman spectra of ZrCl_4_, AlCl_3_, LiF, Li_2_O, LZC, LZACF, and LZACFO. (b) Young's modulus distribution and AFM topography images of LZACFO pellets. (c) SEM image and the corresponding EDS spectrum of LZACFO. (d) HRTEM image of LZACFO.

Raman spectroscopy was employed to probe the vibrational modes and lattice structure of the SEs. As shown in Fig. 2a, a strong and broad band within 120–200 cm^−1^ is observed for LZC, LZACF and LZACFO, corresponding to the F_2g_ bending vibration.^25^ The Raman spectra of LZACF and LZACFO are highly similar to that of LZC, while differing markedly from those of the precursors (ZrCl_4_, AlCl_3_, LiF and Li_2_O), indicating that the mechanochemical process successfully incorporates Al^3+^, F^−^ and O^2−^ into the LZC framework. X-ray photoelectron spectroscopy (XPS) was further conducted to analyze the chemical composition (Fig. S4). The appearance of Al 2p, F 1s and O 1s signals confirms the presence of Al^3+^, F^−^ and O^2−^ in the modified samples. Meanwhile, the positions of the Cl 2p and Zr 3d peaks remain essentially unchanged compared with LZC, suggesting that the cation and anion substitutions do not significantly alter the coordination environment. In the Zr 3d spectrum, the peaks at 183.1 and 185.5 eV correspond to Zr^4+^ 3d_5/2_ and Zr^4+^ 3d_3/2_, confirming the tetravalent state of zirconium.^27^ In addition, the O 1s peak at 530.2 eV can be assigned to Zr–O bonding, further supporting the incorporation of O^2−^ into the lattice.^18^

Highly amorphous SEs typically exhibit enhanced mechanical deformability. Atomic force microscopy (AFM) was therefore used to evaluate the Young's modulus of LZC, LZACF and LZACFO. As shown in Fig. 2b and S5, the average Young's modulus decreases from 32.5 GPa for LZC to 25.9 GPa for LZACF and 15.9 GPa for LZACFO, consistent with the progressive increase in amorphous content. The lower modulus of LZACFO indicates superior mechanical deformability, which is beneficial for achieving intimate interfacial contact between electrolyte particles and cathode active materials.

Scanning electron microscopy (SEM) images (Fig. 2c and S6) show that all samples consist of micrometer-sized particles, while energy-dispersive spectroscopy (EDS) mapping reveals a homogeneous distribution of the constituent elements throughout the particles. Fig. 2d presents the high-resolution TEM (HRTEM) image of LZACFO, revealing nanosized LZC crystallites embedded in an amorphous matrix, confirming the low crystallinity of LZACFO.

**Fig. 3:**
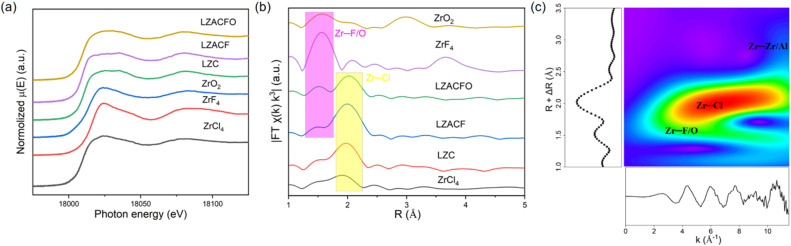
(a) Normalized Zr K-edge XANES spectra and (b) FT of *k*^3^-weighted Zr K-edge EXAFS spectra of ZrCl_4_, ZrF_4_, ZrO_2_, LZC, LZACF, and LZACFO. (c) Wavelet-transformed EXAFS contour plots of LZACFO at Zr K-edge. The original EXAFS signal *χ*(*k*) is weighted by *k*^3^, where *k* represents the wavenumber.

To further probe the local structural characteristics of the amorphous halide SEs, X-ray absorption spectroscopy (XAS) measurements were conducted on LZC, LZACF and LZACFO. As shown in Fig. 3a, the main edge positions of the Zr K-edge X-ray absorption near-edge structure (XANES) spectra for all samples appear at ∼18 020 eV, consistent with those of ZrCl_4_, ZrF_4_ and ZrO_2_, confirming the Zr^4+^ valence state.^40^ Notably, the whiteline features of LZC and LZACF exhibit distinct splitting, whereas LZACFO displays a single, smoother peak, suggesting that oxygen incorporation induces a higher degree of local structural disorder.

The coordination environment around Zr was further investigated by Zr K-edge Fourier-transform EXAFS (FT-EXAFS) spectroscopy (Fig. 3b). The peaks located at ∼2.0 Å and ∼1.5 Å can be assigned to Zr–Cl and Zr–F/O coordination, respectively, based on reference bonds in ZrCl_4_, ZrF_4_ and ZrO_2_. Compared with LZC, LZACFO shows enhanced Zr–O contributions accompanied by reduced Zr–Cl intensity, indicating partial substitution of Cl^−^ by O^2−^ in the Zr coordination environment.

The wavelet-transformed (WT) EXAFS analysis further reveals the coordinating microstructure in the solid state electrolyte. As shown in Fig. 3c, LZACFO exhibits signals corresponding to Zr–O, Zr–Cl and Zr–Zr/Al coordination at approximately 1.5, 2.0 and 3.0 Å, respectively, suggesting the formation of locally disordered [ZrCl_*a*_F_*β*_O_*c*_]^(*a*+*b*+2*c*−4)−^ coordination polyhedra. In contrast, the WT-EXAFS spectrum of LZC shows only Zr–Cl and Zr–Zr signals, consistent with the [ZrCl_6_]^2−^ octahedral coordination (Fig. S7). These results demonstrate that Al^3+^, F^−^ and O^2−^ are incorporated into the Zr coordination environment, generating substantial local disorder and amorphous components. Such non-periodic structures are expected to broaden Li^+^ transport pathways and facilitate rapid Li^+^ migration, consistent with the enhanced ionic conductivity observed in LZACFO.

**Fig. 4:**
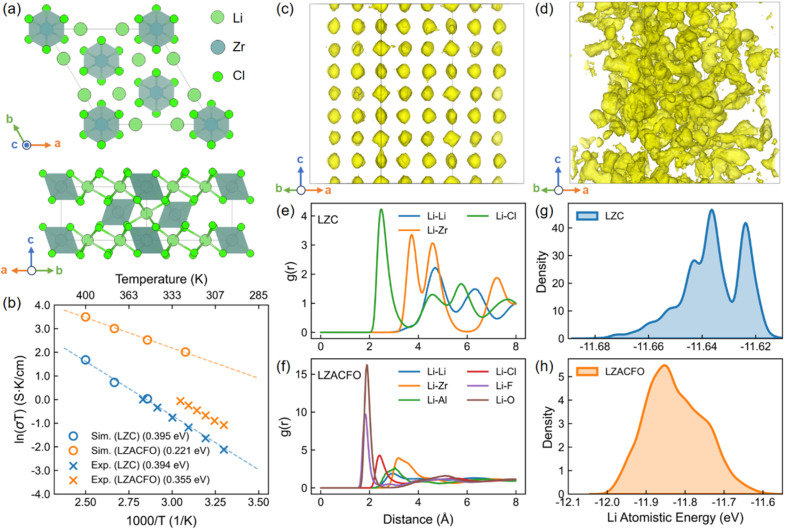
(a) Top and side views of the LZC unit cell. (b) Arrhenius plots of Li-ion diffusion in LZC and LZACFO calculated by machine learning molecular dynamics (MLMD) simulations. (c and d) Three-dimensional Li-ion probability density distributions at 400 K for (c) LZC and (d) LZACFO, rendered at the same isovalue. (e and f) Li-related radial distribution functions extracted from MLMD trajectories for (e) LZC and (f) LZACFO. (g and h) Density of atomistic states of Li-ions in (g) LZC and (h) LZACFO.

To gain deeper atomistic insight into the enhanced Li-ion transport in LZACFO, we performed machine learning molecular dynamics (MLMD) simulations using a high-accuracy neuroevolution potential model with dynamic charges (qNEP)^41^ trained on a comprehensive dataset of 448 structures spanning the configurational space from crystalline LZC to amorphous LZACFO. The qNEP model achieves root-mean-square errors of 4.6 meV per atom, 153.3 meV Å^−1^, and 0.0938 GPa for energies, forces, and stresses, respectively (Fig. S8). The reliability of the qNEP model is validated against the crystalline LZC: as shown in Fig. 4b, the simulated activation energy of 0.395 eV and room-temperature ionic conductivity of 4.27 × 10^−4^ S cm^−1^ agree well with the experimental values of 0.394 eV and 3.97 × 10^−4^ S cm^−1^, respectively, confirming that our qNEP model captures the potential energy surface with high accuracy. For LZACFO, the qNEP model predicts an activation energy of 0.221 eV and a room-temperature conductivity of 1.36 × 10^−2^ S cm^−1^. These values are somewhat higher than the experimental results, which may be partly attributed to the difficulty in fully reproducing the complex compositional and structural heterogeneity of the experimental amorphous phase within idealized simulation models. Nonetheless, the MLMD results clearly reproduce the experimentally observed trend: amorphous LZACFO exhibits substantially lower activation energy and higher ionic conductivity than crystalline LZC.

The Li-ion probability density distributions and radial distribution functions (RDFs) provide direct structural evidence for this difference. At the same temperature and isovalue, the Li-ion probability density in LZC appears highly localized and discontinuous (Fig. 4c), indicating that Li ions are largely confined to well-defined crystallographic sites without forming connected diffusion pathways. In contrast, LZACFO exhibits a much broader and more continuous Li-ion density distribution that extends throughout the simulation cell (Fig. 4d), suggesting the presence of interconnected, three-dimensional diffusion channels enabled by the locally disordered coordination environment. The Li-related RDFs of LZC show a sharp primary peak followed by a series of well-resolved higher-neighbor peaks (Fig. 4e), consistent with the long-range periodic order of the crystalline phase. In LZACFO, only the first nearest-neighbor peak is retained while all higher-order correlations are essentially absent (Fig. 4f), confirming the short-range order but long-range disorder that is characteristic of the amorphous state.

To understand the microscopic mechanisms underlying the structural disorder and its impact on Li-ion diffusion, we employ the density of atomistic states (DOAS) analysis. This approach was first proposed by Wang *et al.* and successfully revealed frustration phenomena in superionic conductors.^42^ This approach is based on the fundamental principle that in machine learning interatomic potentials, the total system energy is obtained by summing individual atomic energies, where each atomic energy is determined by its local atomic environment. By constructing the distribution of atomic energies, DOAS provides direct insight into the variety of local environments and the resulting energy landscape. In crystalline LZC, the Li DOAS exhibits at least two distinct peaks (Fig. 4g), reflecting the presence of multiple inequivalent Li sites with different local environments. In amorphous LZACFO, the discrete peaks merge into a single broad and continuous distribution (Fig. 4h), indicating that Li ions across the amorphous network experience more similar local environments. This much flatter energy landscape lowers the cost of Li-ion hopping between neighboring sites and promotes faster ion migration.

**Fig. 5:**
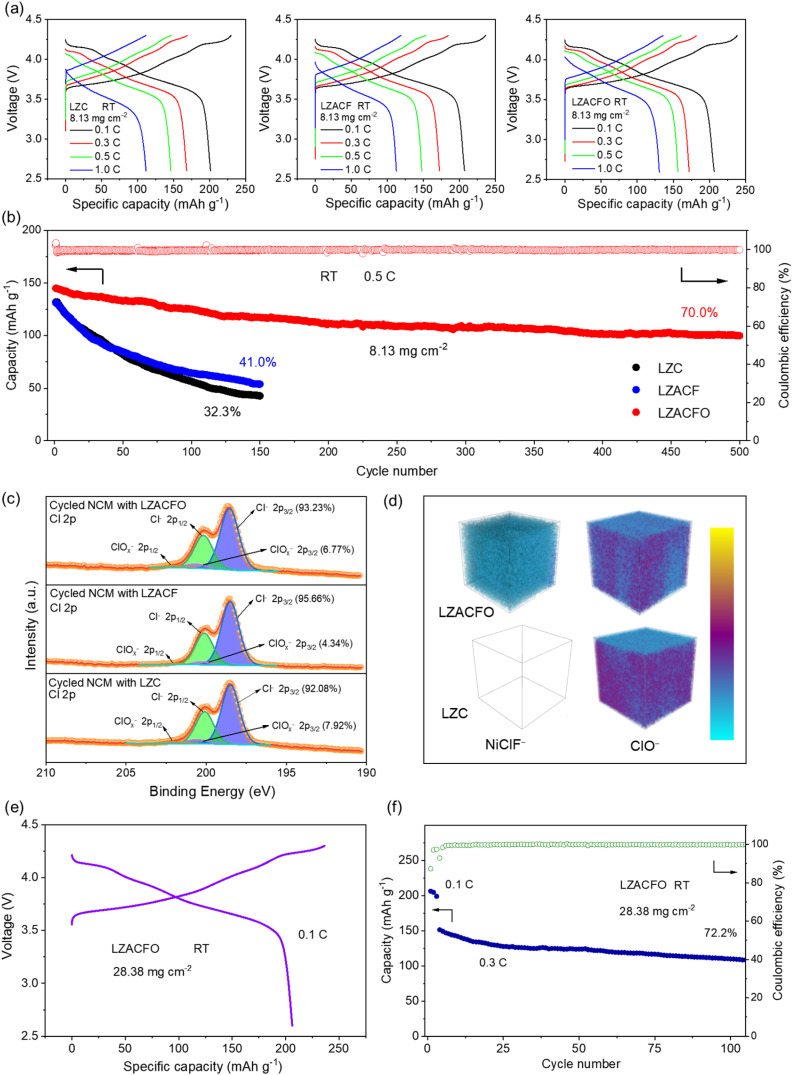
(a) The rate performance of the ASSLBs with LZC, LZACF, and LZACFO. (b) The cycling performance of the ASSLBs with LZC, LZACF, and LZACFO at 0.5C. (c) The deconvoluted Cl 2p XPS spectrum of cycled ASSLBs with LZC, LZACF, and LZACFO. (d) 3D views of the ToF-SIMS analysis of NiClF^−^ and ClO^−^ of cycled ASSLBs with LZC and LZACFO. (e) Initial charge/discharge profiles, and (f) the cycling performance of the ASSLB with LZACFO with the cathode active material mass loading of 28.38 mg cm^−2^.

Following the above characterization, ASSLBs were assembled to evaluate the electrochemical performance of these SEs. A bare LiNi_0.90_Co_0.05_Mn_0.05_O_2_ (NCM90) cathode was employed to construct high-voltage, high-energy-density cells with a configuration of Li–In|Li_6_PS_5_Cl|halide SE|NCM90, operating between 2.6 and 4.3 V *versus* Li/Li^+^ at room temperature. The mass loading of NCM90 in the cathode is 8.13 mg cm^−2^. Fig. 5a presents the galvanostatic charge–discharge profiles of ASSLBs employing different electrolytes. At low current rates (0.1–0.3C), all cells deliver comparable reversible discharge capacities of ∼200 and ∼170 mAh g^−1^, indicating similar initial utilization of the active material. As the current rate increases, differences gradually emerge. At 0.5 and 1C, the LZACFO-based cell exhibits higher capacities of 156 and 131 mAh g^−1^, respectively, compared with LZC (146 and 112 mAh g^−1^) and LZACF (148 and 113 mAh g^−1^), demonstrating its improved rate capability. To further elucidate the origin of the rate differences, galvanostatic intermittent titration technique (GITT) measurements were conducted to probe the reaction kinetics of ASSLBs with different SEs during the initial charge–discharge process (Fig. S9). The effective Li^+^ diffusion coefficients (*D*_Li^+^_) of the LZC- and LZACFO-based cells were calculated (Fig. S10). During the first cycle, *D*_Li^+^_ values for both cells fall within the range of 10^−9^ to 10^−11^ cm^2^ s^−1^. Notably, the LZACFO-based cell exhibits consistently higher *D*_Li^+^_ across nearly the entire voltage range compared to LZC, indicating enhanced Li^+^ transport kinetics. In addition, *in situ* EIS coupled with relaxation time distribution (DRT) analysis was employed to probe the evolution of interfacial stability in the LZACFO-based ASSLB during the first three charge–discharge cycles at 0.1C (Fig. S11). The results indicate a stable electrode–electrolyte interface with negligible interfacial degradation during cycling.

After the rate capability tests (Fig. 5a), the same cells were further cycled to evaluate their cycling stability. As shown in Fig. 5b, at 0.5C, the initial discharge capacities of the LZC-, LZACF-, and LZACFO-based cells are 132, 131, and 145 mAh g^−1^, respectively. During long-term cycling, the LZACFO-based ASSLB shows stable operation over 500 cycles at 0.5C, retaining ∼70% of its initial capacity. In contrast, the LZC- and LZACF-based cells exhibit rapid capacity decay, with retention decreasing to 32.3% and 41.0%, respectively, after only 150 cycles at the same rate, suggesting pronounced interfacial side reactions and structural degradation. Notably, the 500-cycle stability with ∼70% capacity retention surpasses that of most previously reported halide solid electrolytes under comparable conditions (Table S1), highlighting the improved interfacial stability and structural robustness.

To elucidate the origin of the enhanced long-term cycling stability of the LZACF- and LZACFO-based ASSLBs, *ex situ* X-ray photoelectron spectroscopy (XPS) and time-of-flight secondary ion mass spectrometry (ToF-SIMS) depth profiling were performed on cycled composite cathodes to probe the chemical composition of the CEI. As shown in Fig. S12, the deconvoluted Ni 2p spectrum of cycled NCM90 with LZACFO exhibits characteristic peaks of Ni^2+^ 2p_3/2_ (856.1 eV) and 2p_1/2_ (873.6 eV), along with corresponding satellite features, consistent with previous reports.^43^ The absence of Ni^3+^ species is attributed to the fully discharged state of NCM90. Notably, additional peaks at 860.8 and 878.4 eV, assigned to Ni–F bonds,^44^ are observed, indicating the formation of an F-rich CEI. This F-rich interphase effectively stabilizes the cathode surface and suppresses parasitic reactions with the electrolyte, thereby contributing to the improved cycling stability. Fig. 5c compares the Cl 2p XPS spectra of cycled NCM90 with different electrolytes (LZC, LZACF, and LZACFO). The ClO_*x*_^−^ content was quantified as the ratio of the integrated area of the ClO_*x*_^−^ peak to the total integrated area of all fitted peaks in the deconvoluted Cl 2p spectrum. As shown in Fig. 5c, the content of ClO_*x*_^−^ species, arising from the oxidation of chlorine species in the LZACF and LZACFO electrolytes on the surface of NCM90, is 4.34% and 6.77%, respectively. In contrast, the ClO_*x*_^−^ content for the LZC electrolyte on the surface of cycled NCM90 reaches 7.92%. Notably, the ClO_*x*_^−^ content decreases with increasing fluorine content in the electrolytes, indicating that the cathodic stability is effectively enhanced by the introduction of fluorine. This observation is further corroborated by the three-dimensional ToF-SIMS analysis of cycled NCM90 with LZC and LZACFO electrolytes (Fig. 5d). As shown in Fig. 5d, the presence of NiClF^−^ fragments in cycled NCM90 with LZACFO indicates the formation of an F-rich cathode–electrolyte interphase (CEI), whereas the ClO^−^ fragments serve as an indicator of solid electrolyte degradation. Compared with the LZC system, cycled NCM90 with LZACFO exhibits a markedly lower ClO^−^ signal intensity, consistent with the XPS results.

The results shown in Fig. 5c and d indicate that the presence of fluorine effectively enhances interfacial stability, thereby enabling improved cycling performance for cells with LZACF and LZACFO electrolytes compared to those with LZC. Notably, the cell with the LZACFO electrolyte exhibits significantly superior cycling stability relative to that with LZACF, which is attributed to the different amorphous phase contents, as shown in Fig. 1d. Specifically, the increased amorphous content induced by oxygen incorporation reduces the Young's modulus of LZACFO, thereby enabling better accommodation of volume changes during charge/discharge and ultimately leading to enhanced cycling stability.

The above results demonstrate that the LZACFO-based cell exhibits superior cycling stability compared to the other systems, motivating further evaluation of its practical performance. High-energy-density ASSLBs typically require cathodes with mass loadings exceeding 20 mg cm^−2^ and areal capacities of ∼4 mAh cm^−2^. Accordingly, a high-loading cell was constructed using an NCM90 cathode with a mass loading of 28.38 mg cm^−2^. As shown in Fig. 5e, the cell delivers an initial discharge capacity of 206 mAh g^−1^. When cycled at 0.3C, it exhibits an initial discharge capacity of 151 mAh g^−1^ and 72.2% capacity retention after 100 cycles (Fig. 5f).

## Conclusions

In summary, we have developed a multi-anion and cation co-doping strategy to construct a highly amorphous Zr-based halide solid electrolyte (Li_2.25_Zr_0.75_Al_0.25_Cl_4.2_O_0.8_F_0.2_, LZACFO) with enhanced electrochemical performance. The incorporation of O^2−^ significantly increases the amorphous fraction, thereby promoting structural disorder, enhancing mechanical deformability, and facilitating Li^+^ transport. Meanwhile, F^−^ incorporation improves oxidative stability and promotes the formation of a robust cathode–electrolyte interphase. In addition, partial substitution of Zr^4+^ by Al^3+^ increases the Li^+^ concentration *via* charge compensation, further enhancing Li^+^ transport. In addition to enhancing electrochemical performance, Al^3+^ substitution has the potential to reduce electrolyte cost because of the significantly higher abundance and lower cost of Al relative to Zr. The LZACFO-based ASSLB exhibits excellent rate capability and robust long-term cycling performance, retaining ∼70% of its initial capacity after 500 cycles at 0.5C and outperforming most reported halide electrolytes under comparable conditions. This work demonstrates that synergistic multi-anion/cation engineering enables amorphization and Li^+^ enrichment to jointly enhance ion transport while stabilizing electrode–electrolyte interfaces, providing a practical strategy for high-performance halide solid electrolytes.

## Author contributions

Y. Wang and X. Xu conceived the idea and designed the experiments. X. Xu carried out the materials synthesis, characterization and cell testing. Z. Yan and Y. Zhu conducted the calculations. X. Xu, Z. Yan and Y. Wang wrote the draft. Y. Wang supervised the project. All authors discussed the results and commented on the manuscript.

## Conflicts of interest

There are no conflicts to declare.

## Supplementary Material

SC-OLF-D6SC04008J-s001

## Data Availability

The authors confirm that the data supporting the findings of this study are available within the article and its supplementary information (SI). Supplementary information: materials preparation, materials characterization, conductivity measurement, linear sweep voltammetry (LSV) test, electrochemical measurements of ASSLBs, calculation of diffusion coefficients, training dataset construction, qNEP model training, and MLMD simulations. See DOI: https://doi.org/10.1039/d6sc04008j.
