# Supplementary material for: Multi-anion/cation engineering enables fast ion transport and stable interfaces in Zr-based halide electrolytes for all-solid-state batteries
Source: Chem Sci. 2026 Jun 16;17(31):14931–40. doi: 10.1039/d6sc04008j (PMC13297051; doi:10.1039/d6sc04008j)
Supplement: Supplementary file 1 [file SC-017-D6SC04008J-s001.pdf]

Supplementary Information (SI) for Chemical Science.
This journal is © The Royal Society of Chemistry 2026

# Supporting Information

**Multi-Anion/Cation Engineering Enables Fast Ion Transport and Stable Interfaces in Zr-Based Halide Electrolytes for All-Solid-State Batteries**

*Xuele Xu,[a] Zihan Yan,[b] Jiachen Ying,[a] Linghao Deng,[a] Mingxing Liu,[c] Yifei Xu,[c] Yao Liu,[d] Yiwen Zhang,[e] Yizhou Zhu*,[b] Yongyao Xia*[a] and Yonggang Wang*[a]*

*a. Department of Chemistry and Shanghai Key Laboratory of Molecular Catalysis and Innovative Materials, Institute of New Energy, Laboratory of Advanced Materials, iChEM (Collaborative Innovation Center of Chemistry for Energy Materials), Fudan University, Shanghai 200433, China.*

*b. Department of Materials Science and Engineering, Westlake University, Hangzhou, Zhejiang 310030, China.*

*c.State Key Laboratory of Molecular Engineering of Polymers, Department of Macromolecular Science, Fudan University, Shanghai, China.*

*d.*Shanghai Institute of Applied Physics, Chinese Academy of Sciences, Shanghai, 201800, China.

*e.*Mathematics and Science College, Shanghai Normal University, Shanghai 200234, China.

*E-mail: yyxia@fudan.edu.cn (Y.Y. X.), ygwang@fudan.edu.cn (Y. G. W.), zhuyizhou@westlake.edu.cn (Y. Z. Z.)

## Methods

**Materials preparation:** To fabricate LZC, LZCF, LZACF, LZACO, and LZACFO, the raw materials LiCl (99%, Aladdin), $ZrCl_4$ (99.9%, Aladdin), $AlCl_3$ (99%, Aladdin), $Li_2O$ (99.9%, Aladdin), LiF (99.99%, Aladdin) were combined in precise stoichiometric ratios, with each mixture maintaining a total mass of 1 g. The mixture was ground by hand in an agate mortar for 15 minutes to ensure thorough mixing, followed by transferred to a planetary ball mill (Fritsch Pulverisette 7) containing 10 mm $ZrO_2$ balls. Milling was conducted at 400 rpm for 40 h to obtain the final product. To prevent oxidation or hydration, all procedures were conducted in a glovebox flushed with argon gas ($H_2O$, $O_2$ < 0.1 ppm).

**Materials characterization:** X-ray diffraction (XRD) measurements were performed on a Bruker D4 Endeavor X-ray diffractometer with Cu Ka radiation ($\lambda = 1.54178$ Å). To prevent air exposure, an ultrathin Al layer was used to cover the sample holder. Raman spectra were collected on a Raman scattering spectrometer (Horiba LabRAM HR Evolution) equipped with a 532 nm laser. To prevent exposure to air, fluorocarbon rubber gaskets and bolts were used to ensure a tight seal. The morphology and elemental distribution of the samples were examined using a ZEISS GeminiSEM 300 scanning electron microscope (SEM) coupled with energy-dispersive X-ray spectroscopy (EDS) for mapping. High-resolution TEM (HRTEM) was conducted on a JEOL Cryo ARM 300 instrument. X-ray photoelectron spectroscopy (XPS) experiments were conducted using the X-ray photoelectron spectrometer (Thermo Scientific K-Alpha), which was operated at 12 kV and 50 eV using monochromatic Al-Kα radiation. Carbon 1s line at 284.8 eV was used to calibrate. Time of flight secondary ion mass spectrometry (ToF-SIMS) measurements were performed using the time-of-flight secondary ion mass spectrometer (IONTOF GmbH). The area of scanning was 50 μm × 50 μm, while the sputtering area was 300 μm × 300 μm. The Zr K-edge X-ray absorption fine structure spectra were collected on the easy XAFS equipment (easyXAFS 300) under transmittance mode. The spectra were processed and analyzed using the software codes Athena.

**Conductivity measurement:** The ionic conductivity of the materials was evaluated via electrochemical impedance spectroscopy (EIS) obtained by a Bio-Logic electrochemical work station. The 85 mg powder was cold pressed into a 10 mm diameter pellet at 400 MPa and sandwiched between two stainless steel rods. The applied frequency range was 0.1 Hz to 7 MHz, with a voltage amplitude of 10 mV during the ionic conductivity measurement. Ionic conductivity (σ) was calculated using the following equation:

$$\sigma = L/RS,$$

where R is the resistance, L is the thickness, and S is the area of the sample after pressing. Temperature-dependent EIS measurements were conducted at different temperatures. The activation energies of the SEs were calculated using the following equation:

$$\sigma T = \sigma_0 \exp(-E_a/k_B T),$$

where σ is the ionic conductivity, $\sigma_0$ is the Arrhenius prefactor, T is absolute temperature, $E_a$ is the activation energy and $k_B$ is the Boltzmann constant.

**Linear sweep voltammetry (LSV) test:** Conductive carbon (Super P) and SEs were mixed in a mass ratio of 3:7 and ground in an agate mortar for 15 minutes to ensure homogeneity. 60 mg SEs powders were cold-pressed at 360 MPa, followed by 10 mg SEs/Super P composite pressed at 450 MPa. 40 mg $Li_6PS_5Cl$ was homogeneously dispersed on the opposite side of the particles and pressed at 360 MPa. Then, a In foil (10 mm diameter) was attached to the $Li_6PS_5Cl$ surface and a Li foil (1.5 mg) was then attached to the In foil. The cells were pressed at 90 MPa and cycled at a constant external pressure of 85 MPa at 25 °C. All preparation processes were carried out in an argon atmosphere ($H_2O$, $O_2$ < 0.1 ppm). LSV measurements were performed using a Bio-Logic electrochemical work station with a positive scan range from open-circuit voltage (OCV) to 4.3 V versus $Li/Li^+$. The scan rate was 0.1 mV $s^{-1}$.

**Electrochemical Measurements of ASSLBs:** ASSLBs were assembled using LZC, LZACF or LZACFO SEs in combination with a bare $LiNi_{0.90}Co_{0.05}Mn_{0.05}O_2$ (NCM90, Shanshan Tech Co. Ltd) cathode and a Li-In alloy anode. NCM90 was mixed with the halide SE and

carbon nanotubes (CNT) at a 60:38:2 mass ratio. The mixture was ground by hand in an agate mortar for 15 minutes to serve as the cathode composite material. 40 mg $Li_6PS_5Cl$ powder was first placed into a PEEK cylinder (10 mm diameter) and cold pressed into pellets under 360 MPa. Then, 60 mg halide SE was placed on the other side and stacked under 360 MPa. Subsequently, the composite cathode powder was added on one side of the halide SE pellet and pressed at 450 MPa, and the area of cathode is 0.785cm$^2$. Finally, a In foil (10 mm diameter, 55 mg) was attached to the $Li_6PS_5Cl$ surface and a Li foil (1.5 mg) was then attached to the In foil. The ASSLBs were stacked under 90 MPa and tested at a constant external pressure of 85 MPa at 25 °C. All preparation processes were carried out in an argon-flushed glove box ($H_2O$, $O_2$ < 0.1 ppm). Galvanostatic cycling of the ASSLBs was conducted within the voltage range of 2.6 and 4.3 V versus Li/$Li^+$ using LAND battery testing systems.

**Calculation of Diffusion Coefficient:** The effective diffusion coefficient was obtained by GITT. The diffusion coefficient was calculated using the following equation:

$$D_{Li^+} = \frac{4}{\pi\tau}\left(\frac{n_m V_m}{S}\right)^2\left(\frac{\Delta E_s}{\Delta E_t}\right)^2$$

where $\tau$ is the duration of the current pulse, $n_\mathrm{m}$ is the number of moles, $V_\mathrm{m}$ is the mole volume of the electrode, $S$ is the contact area of cathode and SE, $\Delta E_\mathrm{s}$ is the voltage response under the pulse current, and $\Delta E_\mathrm{t}$ is the voltage change during the galvanostatic cycling.

**Training Dataset Construction:** The training dataset was constructed through an active learning workflow implemented in GPUMDkit[1]. The final dataset comprises 448 structures covering a broad range of configurations from crystalline LZC to amorphous LZACFO. All training structures were based on orthorhombic supercells containing 398–432 atoms. Self-consistent field calculations were performed using VASP[2] with the PBEsol exchange-correlation functional[3]. A plane-wave energy cutoff of 520 eV and a Gamma-point-only k-point sampling were adopted, with an electronic energy convergence threshold of $10^{-5}$ eV.

**qNEP Model Training:** We developed the interatomic potentials for LZC and LZACFO using the qNEP model[4]. The radial and angular cutoff radii were set to 6 Å and 5 Å, with the maximum expansion orders for 3-, 4-, and 5-body descriptors being 4, 2, and 1, respectively. To prevent unphysical atomic overlap, the Ziegler-Biersack-Littmark (ZBL) potential[5,6] was incorporated with a global outer cutoff of 2.5 Å and a typewise factor of 0.7. The training converged after $10^5$ iterations (Fig. S8), with other hyperparameters kept at defaults.

**MLMD simulations**: MLMD simulations were performed using *Graphics Processing Units Molecular Dynamics* package[7]. For the ionic conductivity calculations, supercells containing 3456 atoms for LZC and 3184 atoms for LZACFO were used. The MLMD simulations were performed across a temperature range of 325–400 K in the isothermal-isobaric (NPT) ensemble using Martyna–Tuckerman–Tobias–Klein integrators with a timestep of 1 fs. Simulation durations ranged from 1 to 2 ns to ensure sufficient statistical sampling. Additionally, a 500 ps NVT simulation was performed at 400 K for LZC and LZACFO to compute the Li-ion probability density distributions and RDFs. From each NVT trajectory, 100 equally spaced snapshots were extracted for DOAS analysis. All post-processing and analysis were performed using GPUMDkit[1].

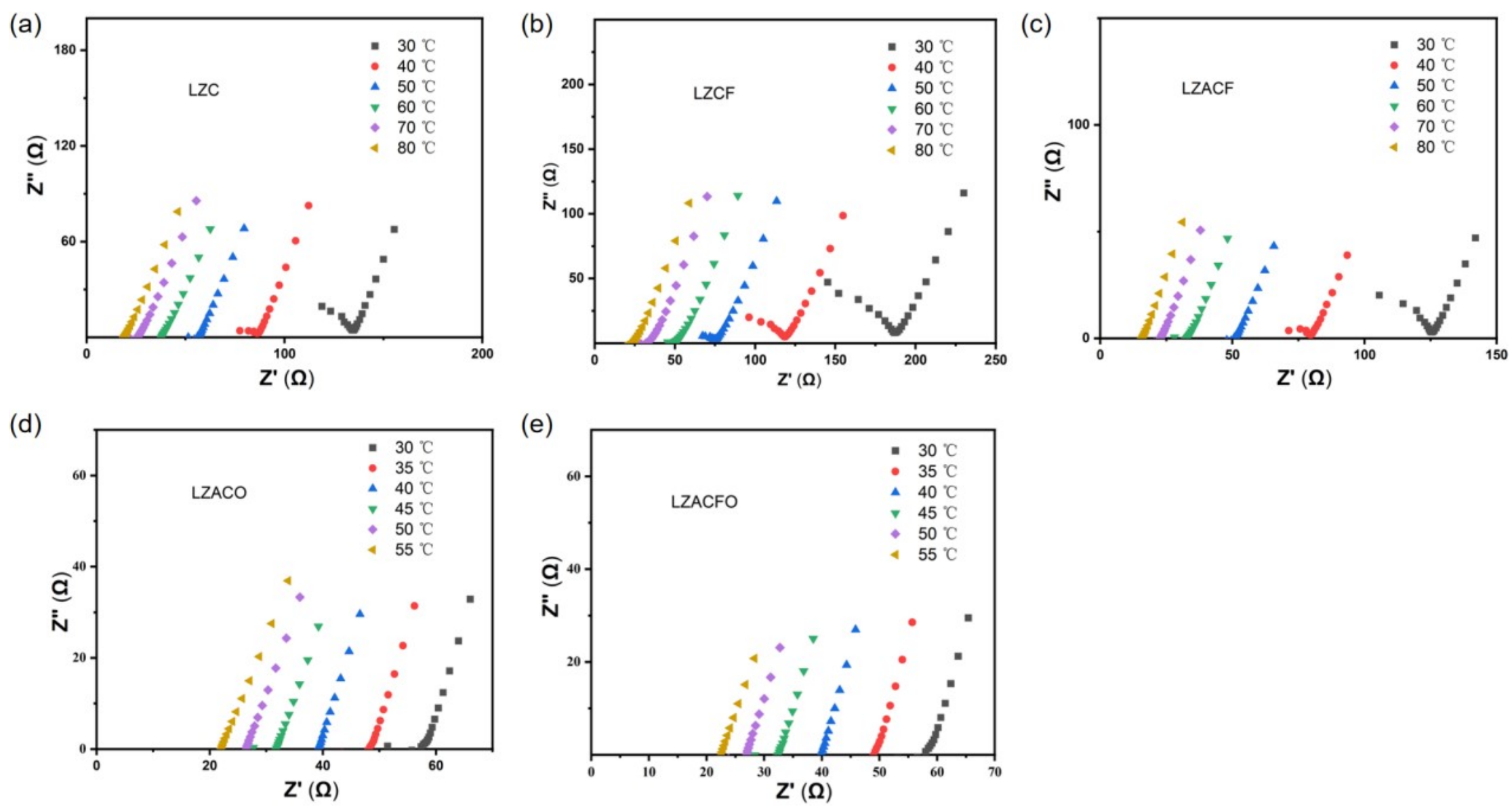


**Figure S1** Nyquist plots of (a) LZC, (b) LZCF, (c) LZACF, (d)LZACO, and (e)LZACFO at different temperatures.

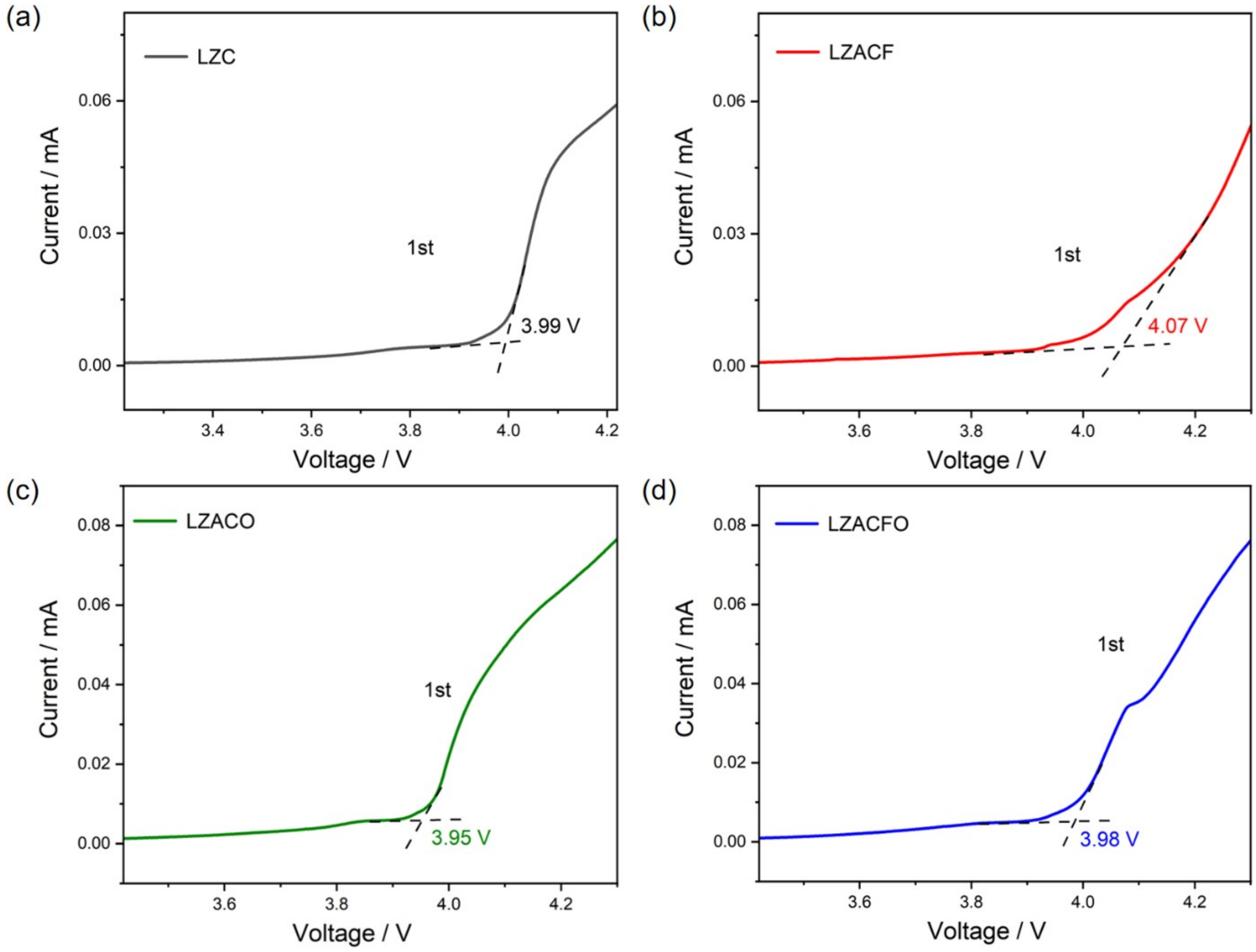


**Figure S2** Scaled-up profiles of the LSV curves of (a) LZC, (b) LZACF, (c) LZACO, and (d) LZACFO corresponding to Figure 1c. Specifically, tangents on either side of the inflection point of the LSV curves were plotted, and the intersection point represented the oxidation potential.

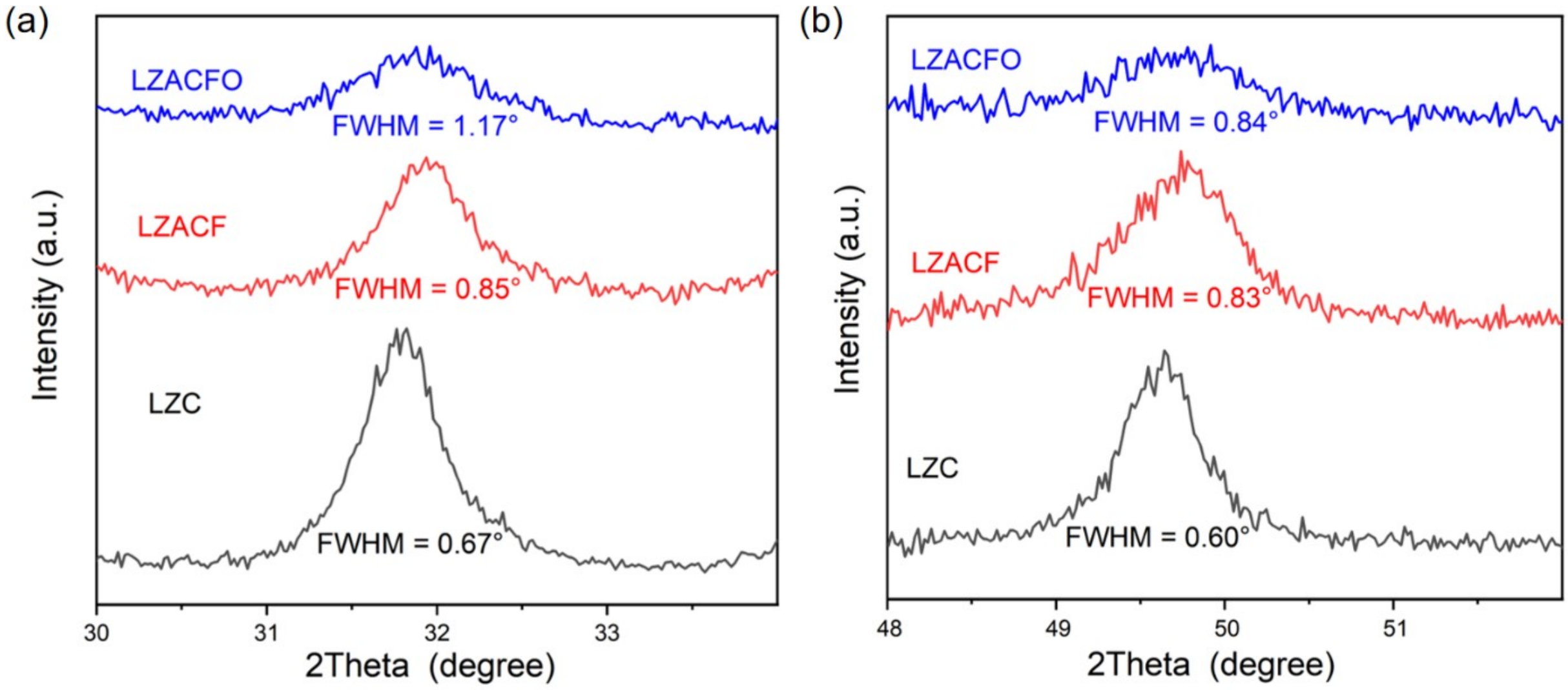


**Figure S3** XRD patterns with magnified regions of (a) 30° to 34° and (b) 48° to 52° of LZC, LZACF, and LZACFO.

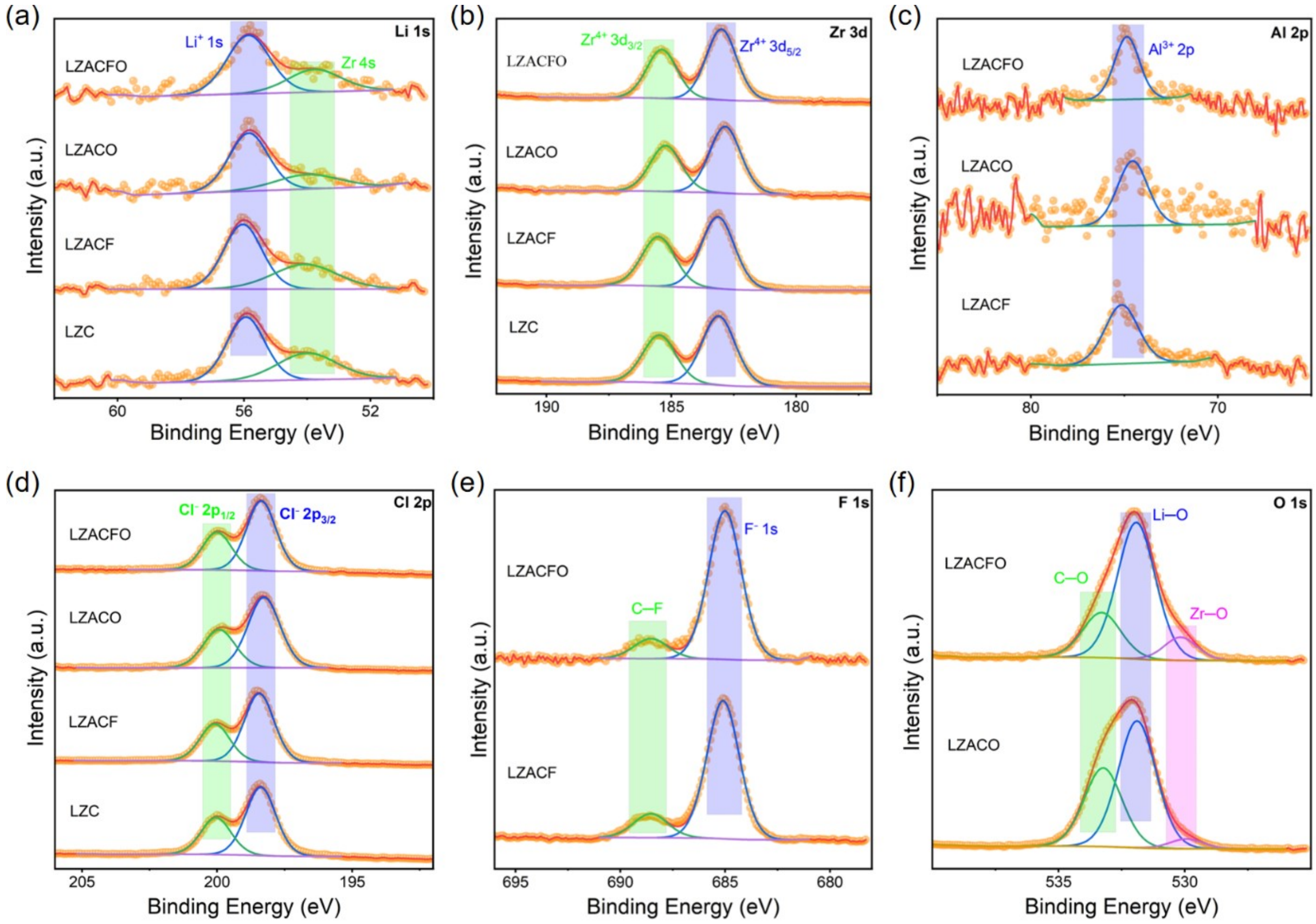


**Figure S4** The XPS spectra of SEs. Deconvoluted XPS (a) Li 1s spectra for LZC, LZACF, LZACO, and LZACFO, (b) Zr 3d spectra for LZC, LZACF, LZACO, and LZACFO, (c) Al 2p spectra for LZACF, LZACO, and LZACFO, (d) Cl 2p spectra for LZC, LZACF, LZACO, and LZACFO, (e) F 1s spectra for LZACF and LZACFO, (f) O 1s spectra for LZACO and LZACFO.

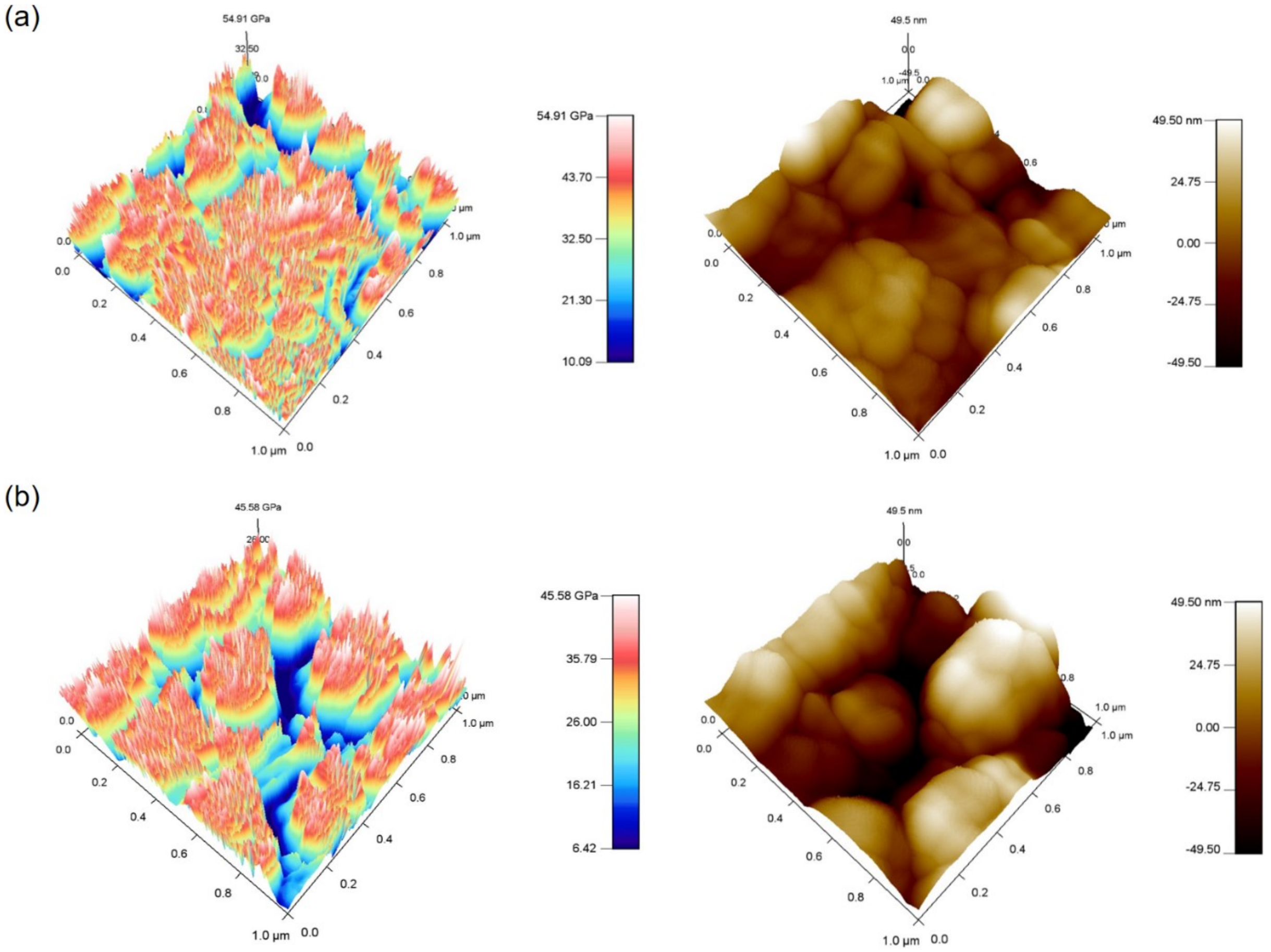


**Figure S5** Young's modulus distribution and AFM topography images of (a) LZC and (b) LZACF pellets.

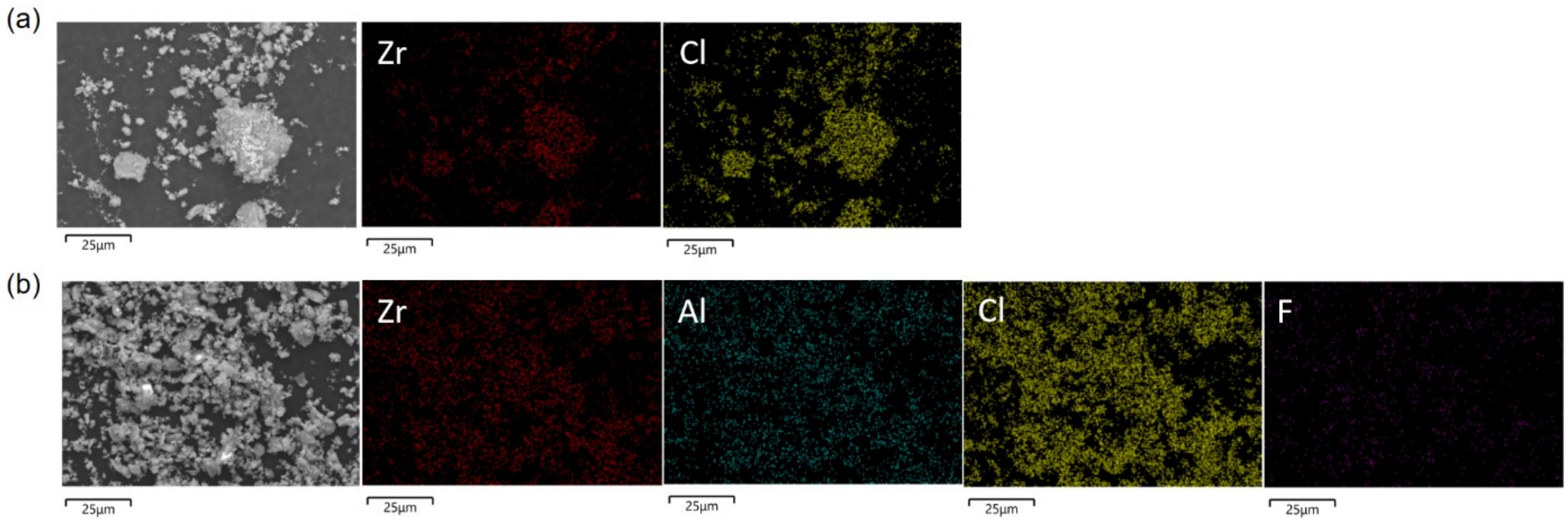


**Figure S6** SEM image and corresponding EDS spectrum of (a) LZC and (b) LZACF.

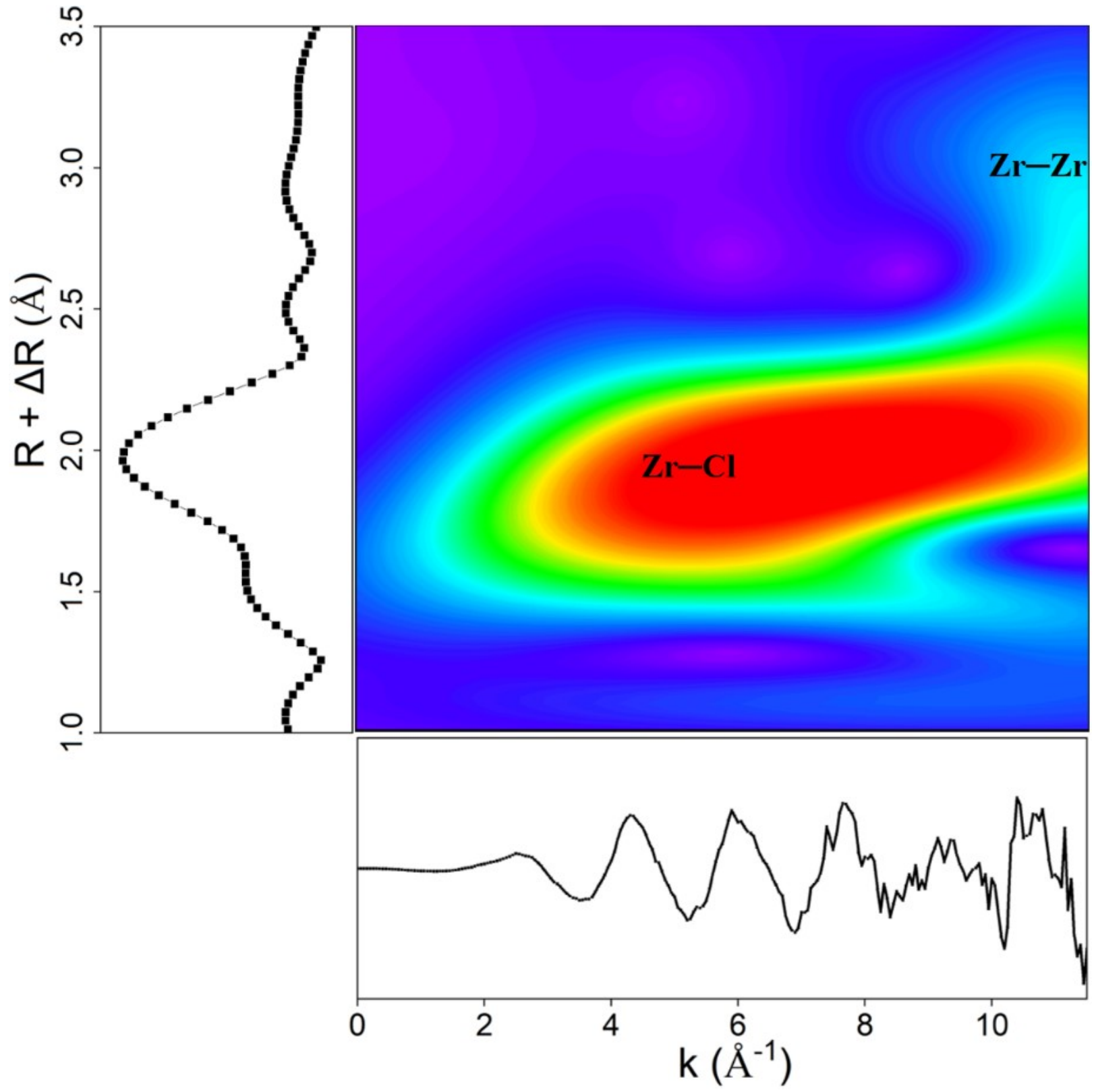


**Figure S7** Wavelet-transformed EXAFS spectrum of LZC at Zr K-edge.

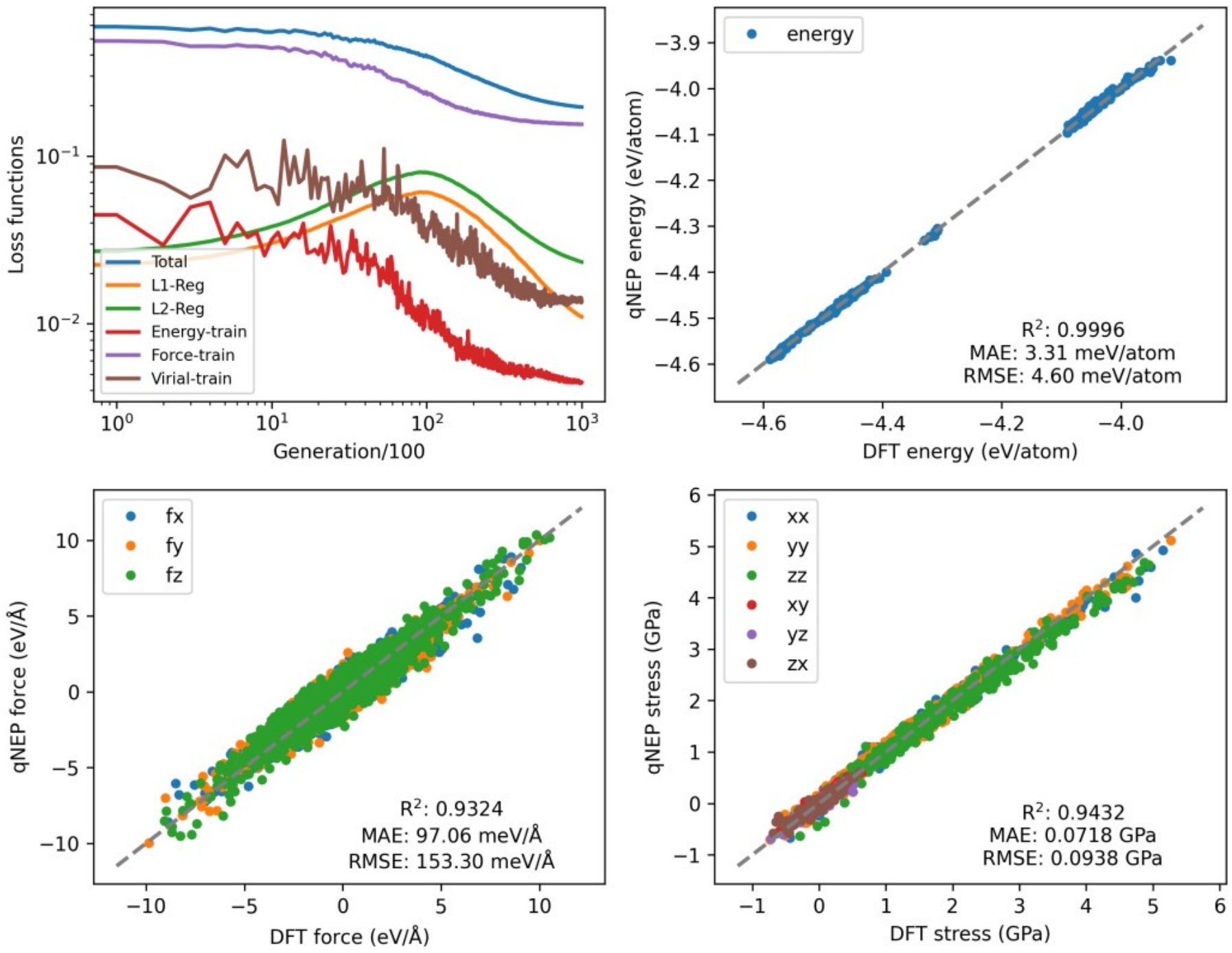


**Figure S8** (a) Evolution of various terms in the loss function during the qNEP training process. (b) Energy, (c) force, and (d) stress values from the qNEP model, in comparison to the DFT reference data.

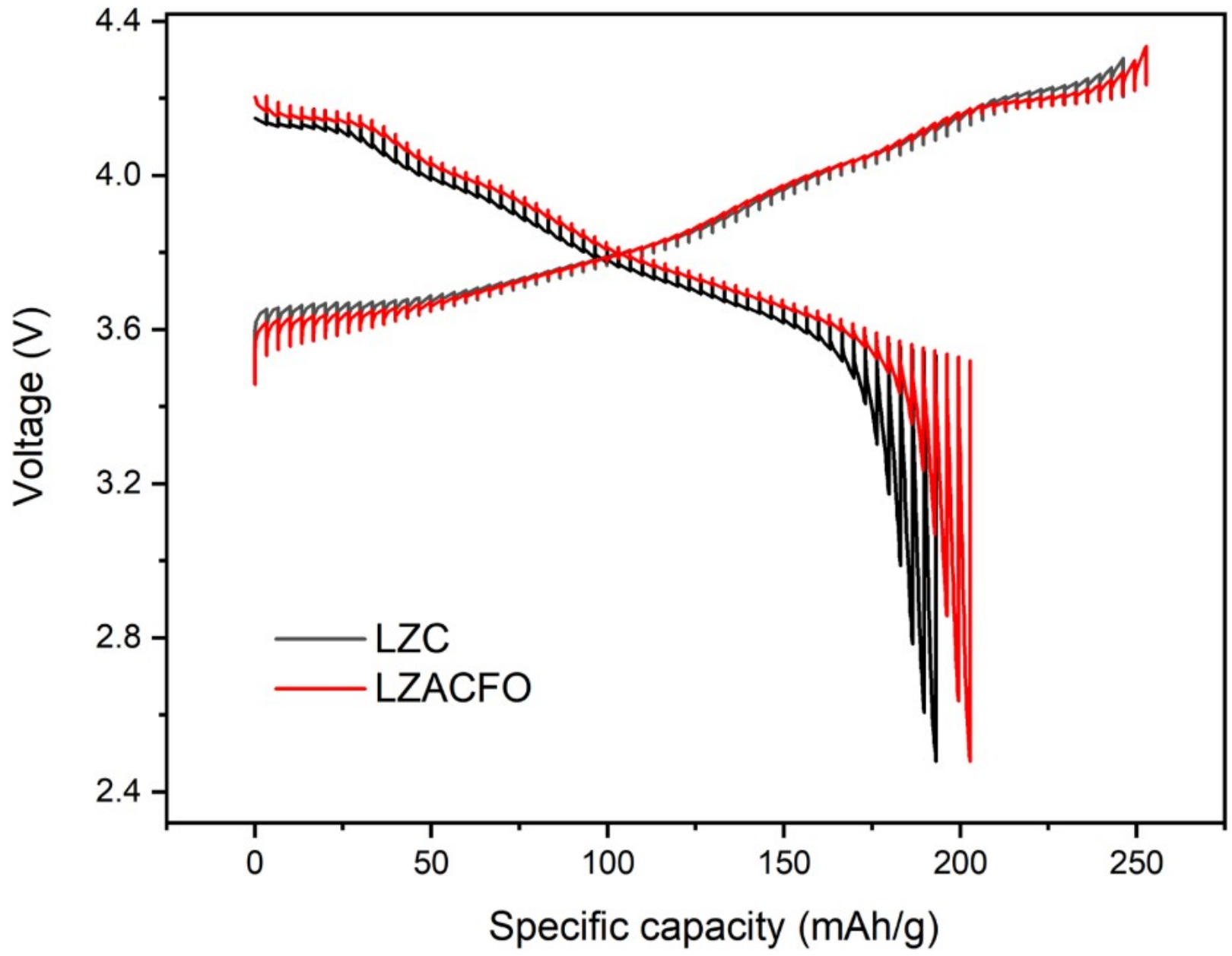


**Figure S9** The GITT profiles of the ASSLBs with LZC and LZACFO for the 1st cycle at 0.1C.

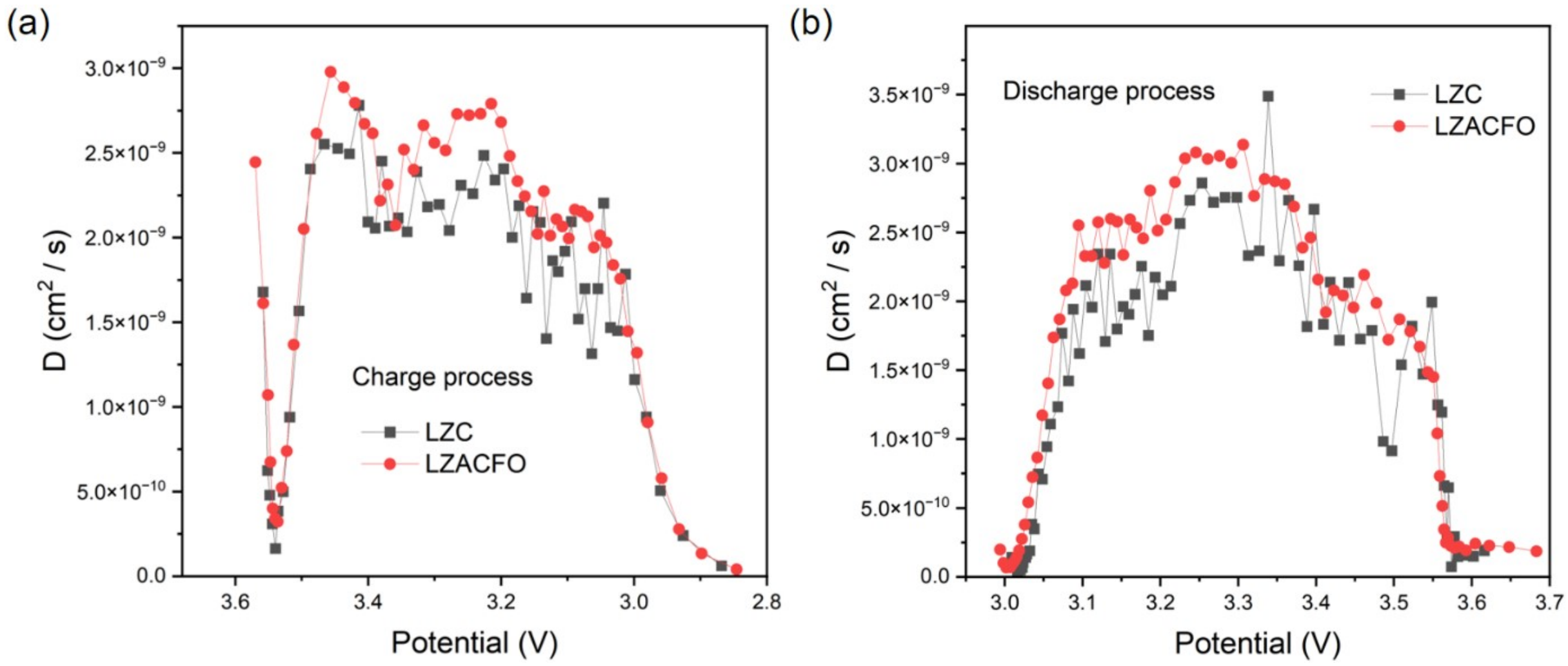


**Figure S10** The effective diffusion coefficient of $Li^+$ of the ASSLB with LZC and LZACFO during (a) charge and (b) discharge processes.

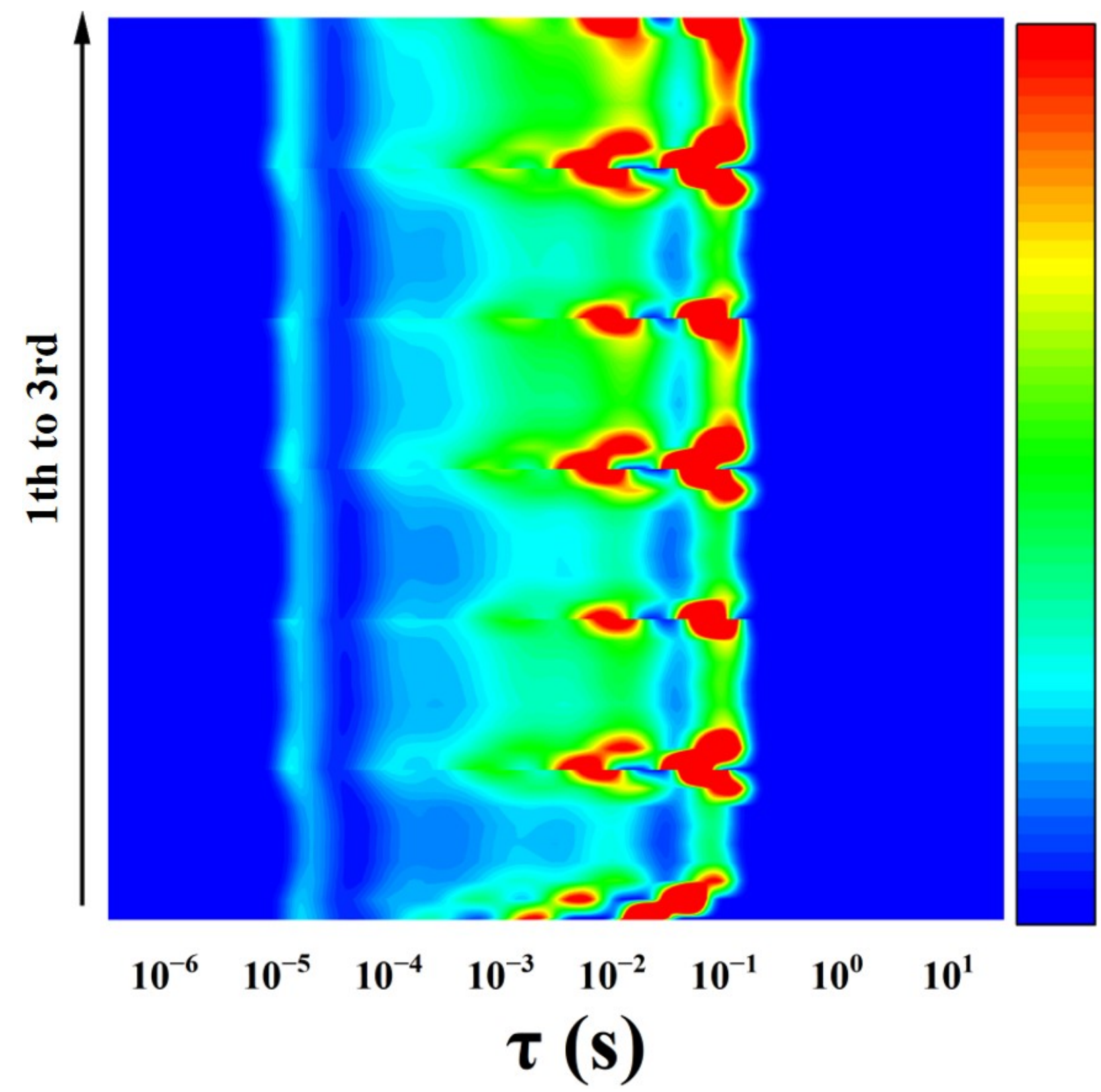


**Figure S11** DRT results for LZACFO during three cycles of charging and discharging at 0.1 C between 2.6 and 4.3 V versus $Li/Li^+$.

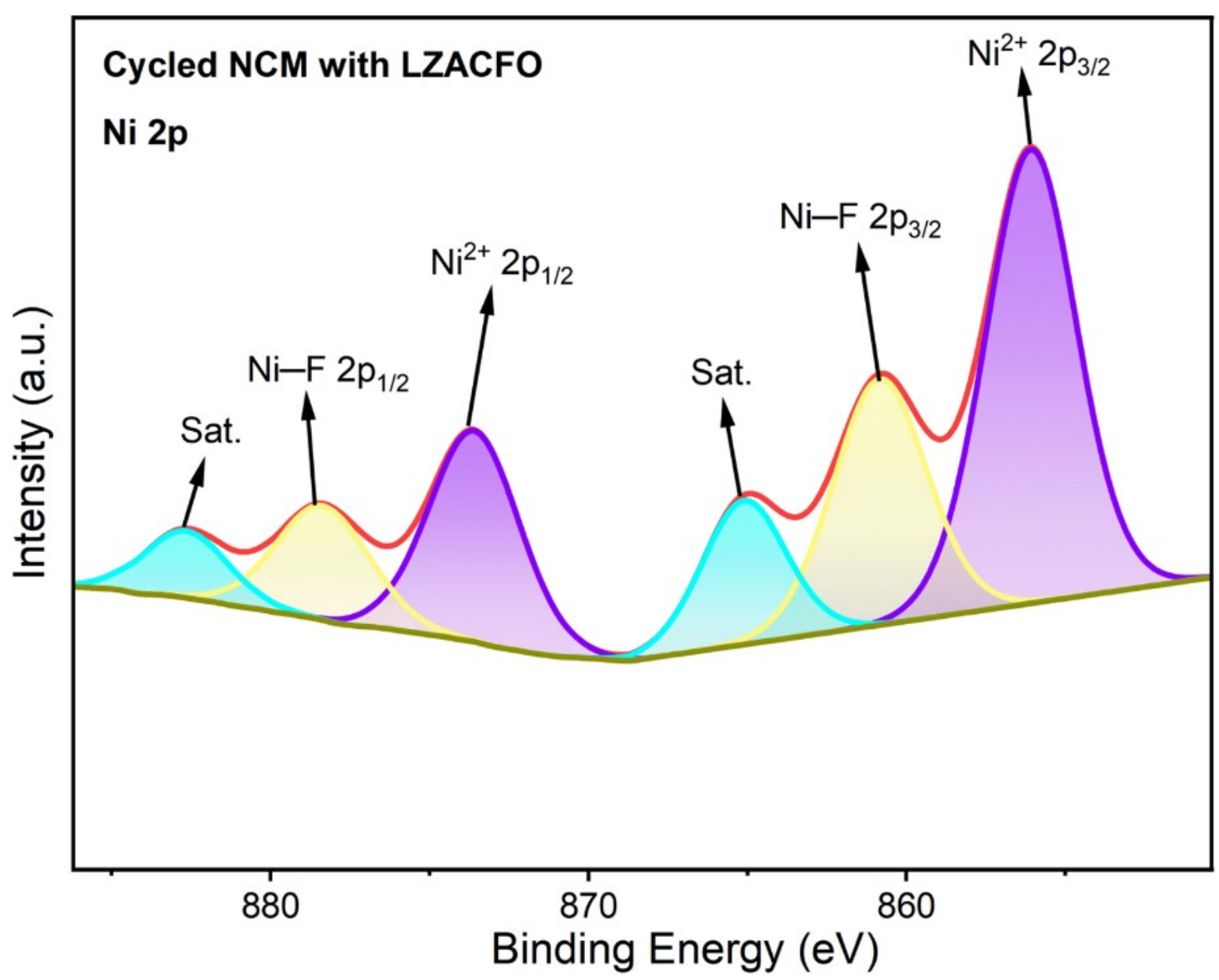


**Figure S12** The deconvoluted Ni 2p XPS spectrum of cycled NCM90 with LZACFO.

**Table S1.** Cycling performance of the LZACOF-based ASSLBs and those based on other halide SEs.

| Ref. | Sample | cathode | Temperature | Rate | Cycle performances |
|---|---|---|---|---|---|
| This work | $Li_{2.25}Zr_{0.75}Al_{0.25}Cl_{4.2}O_{0.8}F_{0.2}$ | NCM90 | 25 °C | 0.5 C | 145 mAh $g^{-1}$, 70.0% retention, 500 cycles |
| Ref 1[8] | $Li_3Fe_{0.1}In_{0.9}Cl_6$ | LCO | NA | 0.2 C | 110 mAh $g^{-1}$, 80.0% retention, 300 cycles |
| Ref 2[9] | $Li_{2.1}Zr_{0.95}Mg_{0.05}Cl_6$ | LCO | 25 °C | 0.3 C | 135 mAh $g^{-1}$, 89.9% retention, 100 cycles |
| Ref 3[10] | $Li_{2.73}Ho_{1.09}Cl_6$ | NMC811 | 25 °C | 0.1 C | 172 mAh $g^{-1}$, 73.0% retention, 180 cycles |
| Ref 4[11] | $Li_{2.7}Yb_{0.7}Zr_{0.3}Cl_6$ | NMC622 | 25 °C | 0.2 C | 170 mAh $g^{-1}$, 80.0% retention, 150 cycles |
| Ref 5[12] | $Li_{2.5}ZrCl_{5.5}O_{0.5}$ | NCM955 | 25 °C | 0.5 C | 130 mAh $g^{-1}$, 56.0% retention, 500 cycles |
| Ref 6[13] | $Li_3ScCl_6$ | LCO | 25 °C | 0.13 mA $cm^{-2}$ | 126 mAh $g^{-1}$, 83.3% retention, 160 cycles |
| Ref 7[14] | $Li_{2.375}Sc_{0.375}Zr_{0.625}Cl_6$ | Ni90 | 30 °C | 0.5 C | 205 mAh $g^{-1}$, 78.1% retention, 200 cycles |
| Ref 8[15] | $Li_{0.538}La_{0.475}Ta_{0.238}Cl_{2.85}O_{0.15}$ | NMC811 | 30 °C | 0.1 C | 184 mAh $g^{-1}$, 70.8% retention, 240 cycles |
| Ref 9[16] | $Li_{2.3}ZrCl_{6.1}F_{0.2}$ | LCO | 30 °C | 0.2 C | 122 mAh $g^{-1}$, 88.3% retention, 150 cycles |
| Ref 10[17] | $Li_{2.25}Zr_{0.75}Al_{0.25}Cl_6$ | NMC811 | 25 °C | 0.1 C | 166 mAh $g^{-1}$, 94.3% retention, 100 cycles |
| Ref 11[18] | $Li_{2.1}Zr_{0.95}Cu_{0.05}Cl_6$ | LCO | 25 °C | 0.2 C | 148 mAh $g^{-1}$, 94.6% retention, 100 cycles |

## Reference

1. Yan, Z. et al., “GPUMDkit: A User-Friendly Toolkit for GPUMD and NEP,” arXiv.2603.17367 (2026).

2. Kresse, G. & Hafner, J., “*Ab initio* molecular-dynamics simulation of the liquid-metal–amorphous-semiconductor transition in germanium,” *Physical Review B* 49 (1994): 14251–14269.

3. Perdew, J. P. et al., “ Restoring the Density-Gradient Expansion for Exchange in Solids and Surfaces,” *Physical Review Letters* 100 (2008): 136406.

4. Fan, Z. et al., “qNEP: A highly efficient neuroevolution potential with dynamic charges for large-scale atomistic simulations,” arxiv:2601.19034 (2026).

5. Liu, J., Byggmästar, J., Fan, Z., Qian, P. & Su, Y., “Large-scale machine-learning molecular dynamics simulation of primary radiation damage in tungsten,” *Physical Review B* 108 (2023): 054312.

6. Yan, Z., Fan, Z. & Zhu, Y., “Improving Robustness and Training Efficiency of Machine-Learned Potentials by Incorporating Short-Range Empirical Potentials,” *Journal of Chemical Information and Modeling* 66 (2026): 1406–1413.

7. Xu, K. et al., “GPUMD 4.0: A high-performance molecular dynamics package for versatile materials simulations with machine-learned potentials,” *Materials Genome Engineering Advances* 3 (2025): e70028.

8. Fu, J. et al., “In-situ formation of stable interface towards Li-in anode for halide solid-state electrolyte,” *Materials Today* 90 (2025): 143–153.

9. Zhang, H. et al., “Li-richening strategy in $Li_2ZrCl_6$ lattice towards enhanced ionic conductivity,” *Journal of Energy Chemistry* 79 (2023): 348–356.

10. Liang, J. et al., “A Series of Ternary Metal Chloride Superionic Conductors for High-Performance All-Solid-State Lithium Batteries,” *Advanced Energy Materials* 12 (2022): 2103921.

11. Kim, S. Y. et al., “Lithium Ytterbium-Based Halide Solid Electrolytes for High Voltage All-Solid-State Batteries,” *ACS Materials Letters* 3 (2021): 930–938.

12. Shen, L. et al., “Anion-Engineering Toward High-Voltage-Stable Halide Superionic Conductors for All-Solid-State Lithium Batteries,” *Advanced Functional Materials* 34 (2024): 2408571.

13. Liang, J. et al., “Site-Occupation-Tuned Superionic $Li_xScCl_{3+x}$ Halide Solid Electrolytes for All-Solid-State Batteries,” *Journal of the American Chemical Society* 142 (2020): 7012–7022.

14. Li, R. et al., “Superionic Conductivity Invoked by Enhanced Correlation Migration in Lithium Halides Solid Electrolytes,” *ACS Energy Letters* 9 (2024): 1043–1052.
15. Yang, J. et al., “$UCl_3$ -Type Crystalline Oxychloride Electrolytes for All-Solid-State Lithium-Ion Batteries,” *Journal of the American Chemical Society* 147 (2025): 36557–36569.
16. Zhang, Y. et al., “A Li-Rich Fluorinated Lithium Zirconium Chloride Solid Electrolyte for 4.8 V-Class All-Solid-State Batteries,” *Small* 21 (2025): 2407418.
17. Gao, K.-N., Bai, F., Sun, Z. & Zhang, T., “Aliovalent substitution of $Al^{3+}$ in $Li_2ZrCl_6$ solid electrolyte towards large-scale application,” *Energy Storage Materials* 70 (2024): 103444.
18. Li, G. et al., “One stone, three birds: $Cu^{2+}$-substituted chloride electrolyte for high-performance all-solid-state lithium batteries,” *Energy Storage Materials* 71 (2024): 103673.
